# A Chinese Girl With LIG4 Syndrome and Hematopoietic Stem Cell Transplantation: A Rare Case Report and Review of the Literature

**DOI:** 10.1002/ccr3.70007

**Published:** 2024-12-17

**Authors:** Chenjia Jiang, Wenyang Wang, Yazhou Li, Xiwang Liu, Zhirui Zhu

**Affiliations:** ^1^ Department of Anesthesiology Children's Hospital, Zhejiang University School of Medicine Hangzhou China; ^2^ Department of Clinical Laboratory Huadong Hospital, Fudan University Shanghai China; ^3^ Department of Cardiac Surgery Children's Hospital, Zhejiang University School of Medicine Hangzhou China

**Keywords:** combined immunodeficiency, DNA ligase 4 syndrome, hematopoietic stem cell transplantation, pediatrics

## Abstract

LIG4 syndrome is an exceptionally rare primary immune deficiency. It is an autosomal recessive genetic disease, falling within the spectrum of genetic disorders characterized by impaired DNA damage response mechanisms. Common clinical characteristics encompass microcephaly, growth retardation, developmental delays, facial deformities, variable immune deficiencies, pancytopenia, heightened susceptibility to malignant tumors, and significant clinical and cellular radiosensitivity. Hematopoietic stem cell transplantation (HSCT) is a curative treatment for LIG4 syndrome and may mitigate the long‐term risk of developing lymphoid malignancies by improving tumor surveillance.

## 1 Introduction

DNA ligase IV (LIG4) deficiency, also known as LIG4 syndrome, is an exceptionally rare primary immune deficiency [1, 2]. It is an autosomal recessive genetic disease, falling within the spectrum of genetic disorders characterized by impaired DNA damage response mechanisms [1]. LIG4 plays a crucial role in the nonhomologous end‐joining mechanism and is essential for repairing DNA double‐strand breaks [3, 4]. In developing lymphocytes, LIG4 is particularly vital for mending programmed DNA double‐strand breaks induced during lymphocyte receptor development [4]. The first report of this condition dates back to 1990, with approximately 50 cases documented worldwide [5, 6]. Sun B et al. [7] provided insights into the distinctive features of DNA LIG4 in a Chinese cohort, including missense mutations (c.833G>T, p. R278L) and deletions such as the c.1271_code shift mutation caused by 1275delAAAGA (p.K424Rfs*20). Common clinical characteristics encompass microcephaly, growth retardation, developmental delays, facial deformities, variable immune deficiencies, pancytopenia, heightened tumor susceptibility, and significant clinical and cellular radiosensitivity [3]. The clinical phenotype of LIG4 syndrome is intricately linked to the mutation's location and type, resulting in substantial diversity in clinical presentations. In 2021, Rashmi Joshi studied that LIG4 mutations confer shorter lifespan in *Drosophila melanogaster* , and LIG4 is required for maintaining health and longevity *Drosophila melanogaster* [8].

## 2 Case Presentation

### 2.1 Case History (Symptoms, Signs, Examinations and Diagnosis)

The baby girl was conceived via in vitro fertilization and born in China. She was full‐term natural childbirth, weighing 1.88 kg. Her parents and sister are healthy without genetic diseases.

At the age of 1, the child was hospitalized for bronchitis, microcephaly, and slow growth and development. The child weighing 5.2 kg, stood at a height of 63 cm, and had a head circumference of 36.5 cm, with an anterior fontanel measuring 0.5 × 0.5 cm. Genetic testing during hospitalization revealed a composite heterozygous variant in the DNA LIG4 gene: c.1271_1275del (p. K424Rfs*19) originating from the mother and c.562C>T (p. R188 W) from the father. These mutations were evaluated as “pathogenic” and “possibly pathogenic,” confirming the diagnosis of LIG4 syndrome.

**FIGURE 1:**
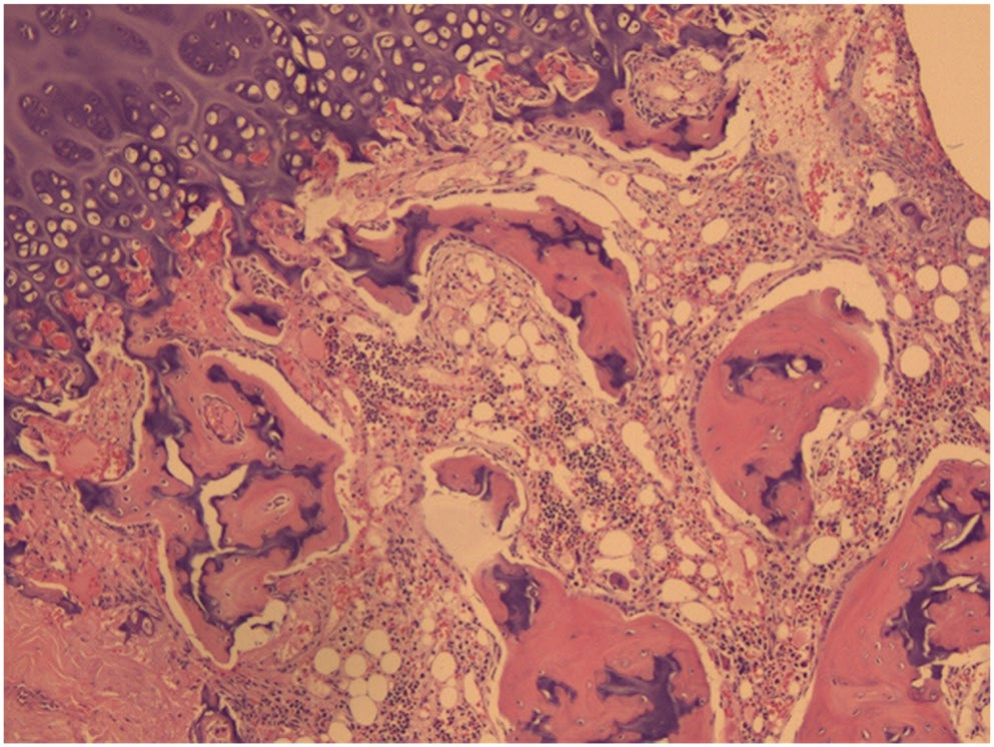
Pathology report (bone marrow aspiration). The ratio of granulocytes to red blood cells was diminished, and there was a significant reduction in granulocytes. Hematopoietic clusters of erythrocytes were visible, while megakaryocytes were rare. Regional fibrous tissue hyperplasia was noted at the myelofibrosis 1 level.

At the age of 2, as the child was preparing to enter kindergarten, a physical examination revealed scattered ecchymosis on both lower limbs. Routine blood tests revealed a white blood cell count of 2.60 × 10^9^/L, hemoglobin at 83 g/L, and platelet count at 37 × 10^9^/L, indicating a need for immediate hospitalization and blood transfusion due to pancytopenia. Bone marrow puncture showed decreased proliferation of hematopoietic tissue, with nucleated cells accounting for approximately 35%. The ratio of granulocytes to red blood cells was diminished, and there was a significant reduction in granulocytes. Hematopoietic clusters of erythrocytes were visible, while megakaryocytes were rare. Regional fibrous tissue hyperplasia was noted at the myelofibrosis 1 level. (Figure 1). When she was 3 years old, hematopoietic stem cells were matched successfully from the Shandong Umbilical Cord Blood Bank (with HLA gene 10/10 matching provider/patient and both having blood type O RhD+/O RhD+).

Physical examination on admission: Body temperature was 36.8°C, heart rate was 112 beats/min, respiratory rate was 24 breaths/min, blood pressure was 92/49 mmHg, head circumference was 40.2 cm, height was 84.5 cm, weight was 8.8 kg, with clear consciousness and good spirits. The child had a small and pale face, soft neck, pharyngeal hyperemia, and thick breath sounds. Heart rate was regular with no pathological murmurs. Her abdomen was soft and the liver and spleen were palpable satisfactorily. There were scattered ecchymoses on both lower limbs, and the limbs were warm with no neurological abnormalities.

Assistant examination: Routine blood tests (Table 1) showed a white blood cell count of 2.07 × 10^9^/L, hemoglobin of 72 g/L, and platelet count of 32 × 10^9^/L. Genetic test revealed a compound heterozygous variant in the DNA LIG4 gene, and bone marrow puncture showed decreased proliferation of hematopoietic tissue.

**TABLE 1:** Blood routine.

	Values (at age 2)	Values (at age 3)	Values (1 Day before HSCT)	Values (1 month after HSCT)	Values (2 months after HSCT)	Values (6 months after HSCT)	Values (1 year after HSCT)	Reference
Leukocyte (10^9^/L)	2.60	1.11	0.20	2.33	3.51	5.46	6.69	4.00–12.00
Lymphocyte (%)	51.2	30.7	18.2	6.2	9.4	10.4	46	20.0–40.0
Neutrophil (%)	32.6	55.7	73.3	62.4	71.5	63.3	34.4	50.0–75.0
Monocyte (%)	11.8	11.89	0.4	1.0.328.9	18.5	24.2	11.8	4.0–16.0
Lymphocyte absolute value(10^9^/L)	1.33	0.34	0.04	0.14	18.5	0.57	3.08	0.70–4.90
Neutrophil absolute value(10^9^/L)	0.85	0.62	0.15	1.46	0.33	3.45	2.29	1.50–7.80
Monocyte absolute value(10^9^/L)	0.31	0.13	0	0.67	2.51	1.32	0.79	0.10–1.50
Red blood cell count(10^12^/L)	2.48	2.26	2.25	3.10	0.65	5.04	5.15	3.50–5.50
Hemoglobin (g/L)	83	72	68	100	147	140	134	110–155
Hematocrit (%)	24.1	20	19.2	30.8	43.7	42.7	40.5	31.0–44.0
Platelet count (10^9^/L)	37	42	76	27	211	196	197	100–400
Plateletcrit (%)	0.40	0.03	0.10	0.03	0.20	0.17	0.18	0.0–20.0
Reticulocyte count (%)	3.5	1.1	0.34	13.40	3.00	2.52	1.31	0.50–1.50

*Note:* The changes of blood routine before and after hematopoietic stem cell transplantation (HSCT).

The child was diagnosed with primary immunodeficiency (LIG4 syndrome), pancytopenia, microcephaly, and slow growth and development.

### 2.2 Treatment and Outcome

The child underwent hematopoietic stem cell transplantation (HSCT). The analysis of chimerism status post‐transplantation revealed that, on Day 7, the provider's blood cells constituted 4.8% of the patient's peripheral blood. On Day 13, this percentage surged to 96.37%, indicating complete chimerism in peripheral blood. The provider's blood cells made up 90.22% of the patient's T cells, displaying mixed chimerism, while the percentage reached 94.02% in the patient's B cells, also indicating mixed chimerism. Furthermore, the provider's blood cells accounted for 99.88% of the patient's NK cells, portraying mixed chimerism. Eventually, the proportion of blood cells derived from the provider reached 97.10%, representing a complete chimeric state. By the 21st day, 99.88% of the provider's peripheral blood cells were entirely chimeric. In the patient's T cells, 99.89% were provider‐derived, and the percentage was 99.93% in the B cells, both reflecting complete chimerism. The patient's NK cells demonstrated 99.90% from the donor blood cells, marking a complete chimeric state. After HSCT, the child experienced post‐transplant complications like infections, fever, and diarrhea. She returned to normal after a month of targeted treatment.

### 2.3 Follow‐Up

Half year after HSCT, and her blood routine reached the normal level (Table 1 shows the changes of blood routine before and after HSCT). Although her height and weight growth have not improved, the child's parents were pleased with the overall medical process, as their primary concern was their child's healthy and happy development.

## 3 Discussion

Congenital immune deficiency constitutes a broad category encompassing over 350 genetic diseases arising from mutations in one or more genes responsible for encoding immune system proteins. DNA repair defects result from mutations in various genes involved in complex nuclear mechanisms tasked with detecting and repairing double‐stranded DNA breaks [1, 3]. DNA constantly endures damage, and if left unrepaired, it can lead to errors in the genetic sequence. These damages can arise from both intracellular events, such as DNA replication and meiosis, as well as extracellular factors, such as damage from reactive oxygen species and ionizing radiation [4]. LIG4, a 911‐amino acid protein containing a DNA binding domain and two BRCT motifs, plays a pivotal role in repairing double‐stranded DNA through the nonhomologous end joining pathway. The nonhomologous end joining is the primary DNA repair mechanism in mammalian cells [3]. LIG4 syndrome is classified under the umbrella of hereditary disorders linked to impaired DNA damage response mechanisms. Clinically and pathologically, patients afflicted with this syndrome typically exhibit microcephaly, distinctive facial features, growth delays, developmental delays, skin anomalies, and a tendency toward pancytopenia. The child had all the above symptoms, and genetic test confirmed this result and diagnosis [3, 9].

Pancytopenia is a common feature in primary immunodeficiency diseases (PIDs), either stemming from bone marrow hematopoiesis suppression or arising secondary to malignant hematological disorders, such as myelodysplastic syndromes [7]. PIDs can manifest as hematological abnormalities, resulting in pancytopenia and, eventually, hematological malignancies. The diagnosis of LIG4 syndrome usually begins with clinical suspicion. Key clinical indicators include microcephaly, combined immunodeficiency (CID) often accompanied by developmental delays, increased vulnerability to bacterial, viral, and fungal infections, leading to multiple hospitalizations, and stunted growth. The most severe form of immunodeficiency is severe combined immunodeficiency (SCID), while rare immunodeficiencies suggest the involvement of defective DNA repair pathways [1, 6, 7]. Additional laboratory features that heighten suspicion include myelodysplasia marked by anemia and thrombocytopenia, lymphopenia with B‐lymphocytopenia, hypogammaglobulinemia, or impairment of isotype classification switching, with elevated IgM and absent or low IgA and IgG [1, 7]. Karyotyping frequently reveals an increase in chromosome 7:14 translocations in many patients, confirming their radiosensitivity. Once radiosensitivity is established, specific genetic testing for DNA LIG4 and other DNA repair genes can be conducted. To date, no cases of DNA LIG4 mutations insensitive to ionizing radiation have been documented [1, 6].

In 2018, Ltmann et al. [1] reviewed 41 cases of DNA LIG4, ranging in age from 2 to 17.5 years, with 68% of the patients being female. Notably, 80% of these patients exhibited microcephaly [3]. She were often hospitalized in the early stage because of bronchitis, microcephaly and slow growth and development. Therefore, she was considered for genetic testing and DNA LIG4 gene was found to be damaged. DNA mutation may be related to in vitro fertilization and embryo transfer. Genetic test revealed a compound heterozygous variant in the DNA LIG4 gene, and bone marrow puncture showed decreased proliferation of hematopoietic tissue. She was diagnosed with primary immunodeficiency (LIG4 syndrome), pancytopenia, microcephaly, and slow growth and development. However, the urgent problem for the child was pancytopenia. The initial treatment for LIG4 syndrome is primarily supportive. It involves providing hematological support for marrow hypoplasia when needed, long‐term administration of antibiotics, antivirals, antifungals, and immunoglobulin substitution [1]. Although HSCT has been employed in a few cases and some studies have reported improvements in platelet counts after intravenous immunoglobulin infusion, it is important to note that conventional immunotherapy is not always effective in treating thrombocytopenia, making HSCT a potential treatment option for LIG4 syndrome [9, 10]. Six months after her treatment, the white blood cells, hemoglobin and platelets of the child (Table 1) had reached the normal level and remained stable. For pancytopenia, HSCT is effective, although her height and weight growth had not improved obviously.

Although everything is fine, her parents still need to be vigilant, she still needs to be regularly observed and followed up. Information on HSCT was available for 10 patients, with successful outcomes in four cases, while the remaining four experienced adverse outcomes. Sadly, four patients did not survive; their causes of death included multi‐organ failure during the conditioning period, Epstein Barr virus‐driven posttransplantation lymphoproliferative disease, and hepatic veno‐occlusive disease. Notably, the use of alkylating agents preceded these unfortunate events. Six patients survived, three of whom received reduced‐intensity conditioning [1, 11]. She reviewed every 3 months until she was seven. HSCT is a curative treatment for CID and SCID immunophenotypes and may mitigate the long‐term risk of developing lymphoid malignancies by improving tumor surveillance. It is crucial that pretreatment regimens exclude irradiation due to the established radiosensitivity of DNA LIG4. Given the systemic nature of DNA LIG4 deficiency, the potential for secondary tumors necessitates an individualized assessment for optimal treatment. Factors such as the patient's immunological profile, infection rate and severity, and dependence on blood products should all be considered in this assessment. It is important to note that HSCT has no effect on microcephaly or neuro developmental delay in LIG4 syndrome patients.

## 4 Conclusion

LIG4 syndrome is a rare genetic disease, and hematopoietic stem cell transplantation is an effective treatment. And HSCT has no effect on microcephaly or neuro developmental delay in LIG4 syndrome patients. Nevertheless, HSCT can lead to many complications and adverse consequences, which require our active management.

## Author Contributions

**Chenjia Jiang:** conceptualization, data curation, investigation, methodology, writing – original draft, writing – review and editing. **Wenyang Wang:** methodology, validation, writing – original draft, writing – review and editing. **Yazhou Li:** data curation, formal analysis, investigation, resources, software, writing – original draft, writing – review and editing. **Xiwang Liu:** investigation, methodology, project administration, supervision, validation, writing – original draft, writing – review and editing. **Zhirui Zhu:** formal analysis, funding acquisition, resources, software, writing – original draft, writing – review and editing.

## Ethics Statement

The patient was treated in accordance with the Declaration of Helsinki and has provided informed consent for the treatment/procedures, and consent for publication.

## Consent

Written informed consent was written by the parents of our patient to participate in this study.

## Conflicts of Interest

The authors declare no conflicts of interest.

## Data Availability

The datasets generated and analyzed during this study are available from the corresponding author upon reasonable request.
